# Surgical Timing in Thyroid Cancer with Lateral Neck Metastases: Delayed Versus Contemporary Lateral Neck Dissection

**DOI:** 10.3390/cancers17162649

**Published:** 2025-08-14

**Authors:** Francesco Chu, Rita De Berardinis, Marta Tagliabue, Roberto Bruschini, Stefano Filippo Zorzi, Marco Federico Manzoni, Maria Cecilia Mariani, Enrica Grosso, Gioacchino Giugliano, Mohssen Ansarin

**Affiliations:** 1 Department of Otolaryngology and Head and Neck Surgery, European Institute of Oncology (IEO), IRCCS, Via Ripamonti 435, 20141 Milan, Italy; francesco.chu@ieo.it (F.C.); roberto.bruschini@ieo.it (R.B.); stefano.zorzi@ieo.it (S.F.Z.); enrica.grosso@ieo.it (E.G.); gioacchino.giugliano@ieo.it (G.G.); mohssen.ansarin@ieo.it (M.A.); 2 Onco-Endocrinology Unit, European Institute of Oncology (IEO), IRCCS, 20141 Milan, Italy; marcofederico.manzoni@ieo.it (M.F.M.); mariacecilia.mariani@ieo.it (M.C.M.)

**Keywords:** thyroid cancer, neck metastasis, lateral neck dissection, vocal palsy, complication

## Abstract

**Backgrounds.** Lateral neck dissection (LND) is standard for thyroid cancer patients with neck metastases, mostly performed at the same time as total thyroidectomy (cLND). We introduced a new delayed LND (dLND), 4 weeks after thyroidectomy to reduce surgical morbidity. This study aims to compare the oncologic/complication outcomes between the two strategies, based on a large retrospective cohort of patients. **Methods.** Between 1996 and 2024, 215 patients were treated with total thyroidectomy, central neck dissection (CND) and LND, and grouped by surgical strategy (cLND vs. dLND); survival/complication outcomes were analyzed and compared between the two groups. **Results.** The overall and disease-free survival were comparable between groups. Age, extracapsular extension, and nodal burden predicted recurrence. dLND was associated with a significantly lower risk of vocal fold palsy. Extranodal extension (ECE) strongly predicted nerve injury. **Conclusions.** dLND offers similar oncologic outcomes to cLND, with reduced risk of vocal fold palsy. A staged approach enhances nerve preservation and might be considered in treatment planning.

## Simple Summary

Most patients with differentiated thyroid cancer have an excellent prognosis after treatment. In patients without lymph node involvement (pN0), surgery is usually limited to the thyroid, and lateral neck dissection is not required. Conversely, in patients with lateral neck metastases (pN1b), surgery is typically extended to include lymph node removal in a single, comprehensive procedure. However, performing everything at once may increase the risk of complications such as vocal cord paralysis. In this study, we compared two surgical strategies: removing the lymph nodes during the initial operation or delaying that step to approximately four weeks later. We found that the delayed approach offered the same excellent cancer outcomes but significantly reduced the risk of permanent voice changes. These findings suggest, for the first time, that a two-step procedure may be a safer option for selected patients, particularly those with additional health conditions, and should be considered in personalized treatment planning.

## 1. Introduction

Thyroid cancer has shown an increasing incidence trend in the United States over the past two decades, with an estimated 53,000 new cases diagnosed in 2020. More recently, the incidence rate has stabilized, likely due to more conservative criteria for thyroid biopsy and the reclassification of non-invasive follicular thyroid neoplasm with papillary-like nuclear features [1,2].

Surgery remains the primary treatment for differentiated thyroid tumors, with nearly 200,000 thyroidectomies performed annually in the United States [3,4]. Prognosis is generally favorable, with excellent long-term outcomes. However, postoperative complications are not negligible and vary widely in the literature, depending on the extent of surgery and the need for lymph node dissection [5,6,7].

Several authors have raised concerns about the association between lymph node dissection and surgical morbidity. At the European Institute of Oncology, we previously showed that central neck dissection (CND), when performed in both prophylactic and therapeutic contexts, does not increase the risk of complications [8,9,10]. In contrast, lateral neck dissection (LND) is typically reserved for patients with confirmed nodal metastases on preoperative evaluation due to its higher risk of postoperative sequelae—including transient or permanent hypoparathyroidism, recurrent laryngeal nerve injury, and more severe events such as bleeding, hematoma, or airway compromise [5,7,11,12].

Improving our understanding of risks associated with LND is essential for guiding preoperative counseling and surgical planning. Given the generally excellent prognosis of differentiated thyroid cancer and the long-life expectancy of most patients, it is reasonable to consider alternative treatment strategies that reduce the risk of long-term morbidity.

Although LND remains the gold standard procedure when resectable lateral neck metastases are present, in our institution, an alternative strategy was adopted in selected cases, based on the clinical perception that a single-stage procedure might carry a higher risk of postoperative complications in certain patients.

At our department (European Institute of Oncology, IRCCS, Milan), we have treated over 100 thyroid cancer cases annually for the past two decades, and starting in 2013, a delayed approach to lateral neck dissection (dLND), performed 3–4 weeks after total thyroidectomy and CND, was selectively adopted for cN1b patients with relevant comorbidities. This individualized strategy emerged in response to clinical concerns about increased rates of postoperative complications observed in patients undergoing extensive one-stage surgery. Although no formal protocol or publication had yet codified this approach, it was shaped by shared institutional experience and institutional multidisciplinary discussions. The underlying hypothesis was that a two-step surgical plan might help reduce overall surgical trauma and complications in fragile patients, without compromising oncological outcomes.

In the present study, we compare—for the first time—oncological outcomes (survival rates) and surgical complications between two treatment strategies for cN1b differentiated thyroid cancer, total thyroidectomy with CND and contemporary LND (cLND) versus total thyroidectomy with CND followed by dLND, using rigorous statistical adjustment techniques, including Inverse Probability of Treatment Weighting (IPTW).

## 2. Materials and Methods

From January 1996 to June 2024, about 4000 patients underwent surgical treatment for thyroid cancer at the Department of Otolaryngology—Head and Neck Surgery, European Institute of Oncology (IEO) IRCCS, Milan, Italy. This retrospective cohort study included patients who underwent total thyroidectomy, CND, LND, and postoperative radioactive iodine (RAI) therapy for differentiated thyroid cancer.

All patients were evaluated preoperatively with fine-needle aspiration cytology and treated by surgeons with a minimum of five years of experience, following the standardized anatomical surgical protocol used at our institution [8,9,10]. LND was routinely performed from level II to level V. From 1996 to 2012, N1b patients were predominantly treated with one-stage surgery. Beginning in 2013, following an internal review and based on the good prognosis of papillary thyroid cancer, a two-stage procedure was introduced for selected cN1b patients with significant comorbidities. This approach included total thyroidectomy with CND followed by dLND, typically performed 4–6 weeks after the initial operation.

All surgical procedures were performed using 3.5× magnification loupes by experienced head and neck surgeons with specific expertise in thyroid cancer. Intraoperative neuromonitoring was not routinely employed but was selectively adopted in high-risk cases, particularly those with extensive extranodal spread, extracapsular extension, or massive tumor infiltration involving both thyroid and nodal compartments (“T-N” axis). In these settings, neuromonitoring was used to support nerve identification and preservation during complex dissections.

During the preoperative evaluation, all patients underwent a complete clinical examination, flexible laryngoscopy to assess vocal cord mobility, thyroid and neck ultrasound (US) for nodal assessment, and standard laboratory tests including serum calcium.

Clinical and pathological records were reviewed independently by two experienced head and neck surgeons (F.C. and R.D.B.). The following data were collected: age at surgery, sex, Charlson Comorbidity Index (CCI), body mass index (BMI), final pathological T and N stages (pT and pN) according to the 8th AJCC Classification of Thyroid Cancer [13], number of metastatic lymph nodes (at level VI and levels II–V), total number of excised lymph nodes, multifocality, extracapsular extension (ECE), and thyroidectomy and neck dissection dates.

Postoperative complications were also recorded, including vocal cord palsy, hypocalcemia, wound infection, postoperative bleeding, chyle leak, shoulder syndrome, and Horner’s syndrome.

Vocal fold palsy and hypocalcemia were further classified as transient and permanent if they lasted more or less than 6 months [14,15,16].

Patients were excluded if distant metastases were present at the time of diagnosis.

Follow-up data were collected to assess oncological outcomes, including overall survival (OS), disease-free survival (DFS), and disease-specific survival (DSS).

When this surgical strategy was introduced, the choice between cLND and dLND followed a cautious internal decision-making process that combined clinical judgment with multidisciplinary input. All patients underwent a thorough preoperative workup, including anesthesiology evaluation, and their surgical risk profile was reviewed during multidisciplinary board discussions. dLND was generally considered for patients with significant comorbidities or increased frailty, based on the idea that staging the procedures could help reduce immediate postoperative complications. Although this approach required two separate anesthesia sessions, each procedure was shorter and often better tolerated. Disease-related factors were also taken into account, such as the number and size of suspicious lymph nodes, absence of extranodal extension or major soft tissue invasion on imaging (ultrasound or CT), and the feasibility of a safe second-stage dissection.

Institutional Review Board approval was obtained (IEO code 2044), and patient informed consent was waived due to the retrospective nature of the study. Nonetheless, at the time of surgery, all patients were thoroughly informed about the available surgical strategies. For those eligible for dLND, the alternative between a one-stage and a two-stage approach was discussed in detail. Patients were made aware that the delayed strategy, although not yet formally validated, was being selectively adopted in our center based on strong institutional experience suggesting a potential reduction in postoperative complications. The staged approach was proposed as an alternative option, and patient preference was always incorporated into the final surgical decision.

### 2.1. Statistical Analysis

Continuous variables were summarized as median and interquartile range (IQR) and compared using the Mann–Whitney U test. Categorical variables were reported as counts and percentages and compared with the χ^2^ test or Fisher’s exact test, as appropriate.

Overall survival (OS) and disease-free survival (DFS) were estimated using the Kaplan–Meier method and compared with the log-rank test. Ninety-five percent confidence bands were omitted for clarity. Time-to-event outcomes were measured from the date of primary surgery. Patients were censored at the date of last known follow-up or death from unrelated causes, whichever occurred first.

Cox proportional hazards regression was used to estimate adjusted hazard ratios (HRs). Candidate covariates for univariate analysis included age at surgery, sex, Charlson Comorbidity Index (CCI), pathological T stage, tumor multifocality, number of metastatic lymph nodes (levels VI and II–V), and extracapsular extension (ECE). For multivariate analysis, to minimize overfitting, we adhered to the rule of 10 events per degree of freedom and selected the most significant predictors from the univariate analysis.

Crude comparisons of postoperative complications between contemporaneous and delayed lateral neck dissection (cLND vs. dLND) were expressed as risk ratios (RRs) with Wald 95% confidence intervals. Due to low cell counts for some complications, Fisher’s exact test was used where appropriate.

To address potential confounding, we applied the inverse probability of treatment weighting (IPTW) based on propensity scores derived from logistic regression. For survival analyses, the model included age, sex, CCI, pathological T stage, ECE, multifocality, and the number of pathologic lymph nodes in levels II–V and VI. For complication analysis, the IPTW model included age, sex, pathological T stage, multifocality, ECE, and level VI nodal burden. Covariate balance was assessed using standardized mean differences (SMD), with values < 0.10 considered indicative of adequate balance.

As a sensitivity analysis, we also performed 2:1 propensity score matching based on the same covariates. Matched results were consistent with the IPTW findings and are reported in the Supplementary Materials.

### 2.2. Model Diagnostics and Robustness Checks

For the survival models, proportional hazards assumptions were tested using scaled Schoenfeld residuals (global and covariate-specific). No significant violations were observed. Due to a declining baseline hazard, a Weibull accelerated failure time (AFT) model was fitted as a sensitivity check, incorporating a 0.01-month offset for one DFS observation with zero duration; the AFT estimates were concordant with those from the Cox model, supporting robustness.

For the complications analysis, model diagnostics were conducted on IPTW-weighted logistic regression models. Discrimination and calibration were evaluated using the area under the receiver operating characteristic curve (AUC), weighted Brier score, and Hosmer–Lemeshow goodness-of-fit test.

Additional sensitivity analyses included IPTW weight trimming at the 1st–99th and 5th–95th percentiles, doubly robust estimation, and 1000-bootstrap resampling for bias-corrected confidence intervals. Influence diagnostics (DFBETAs) confirmed that no single observation had a disproportionate effect on the main predictors. Finally, E-values were calculated to assess the potential impact of unmeasured confounding.

All analyses were conducted using Python 3.11 (Python Software Foundation), employing standard packages for statistical modeling, including lifelines, statsmodels, and scikit-learn. A two-sided *p* < 0.05 was considered statistically significant.

### 2.3. Calendar Date Bias Correction

Considering the long follow up time, we decided to exclude the effect of potential calendar date bias such as surgical learning curves or differences in operating surgeons by conducting a separate analysis in a more recent cohort (2013–2024). Thus, we performed all the statistical analyses in a smaller and recent cohort of representative patients treated between 2013 and 2024.

## 3. Results

The clinical and pathological characteristics are summarized in Table 1.

From January 1996 to June 2024, 260 out of 3685 thyroid cancer patients were treated in our institution for pT1-4 pN1b differentiated thyroid cancer. Seven patients were excluded for distant metastases at diagnosis (lung, liver, bone). Focusing on the pT stage, 38 patients were affected by pT4 cancer. Due to the strong imbalance due to the vast majority of pT4 cases treated in the early years with contemporary neck dissection, it was not possible to extend the analysis to pT4; thus, the final cohort included 215 patients with thyroid cancer and lateral neck metastases who underwent total thyroidectomy with CND and LND.

The mean age at surgery was 42.8 ± 15.1 years for the cLND group and 45.6 ± 15.2 for the dLND group, with a female predominance. Median follow-up was 93 months (IQR: 28–152 months) providing robust long-term outcome data.

Most patients had early-stage primary tumors, 155 patients with pT1 disease (72.1%), 37 with pT2 (17.2%), and 23 with pT3 (10.7%). Overall, 122 out of 215 patients (≈56.7%) had multifocal differentiated thyroid cancer. Extracapsular nodal spread was identically found in 122 of 215 patients (≈56.7%).

The dLND group included 79 patients (36.7%), while the cLND included 136 patients (63.3%). For the delayed group, the median time between surgeries was 39 days (IQR: 30–51).

### 3.1. Survival Outcomes

**Figure 1:**
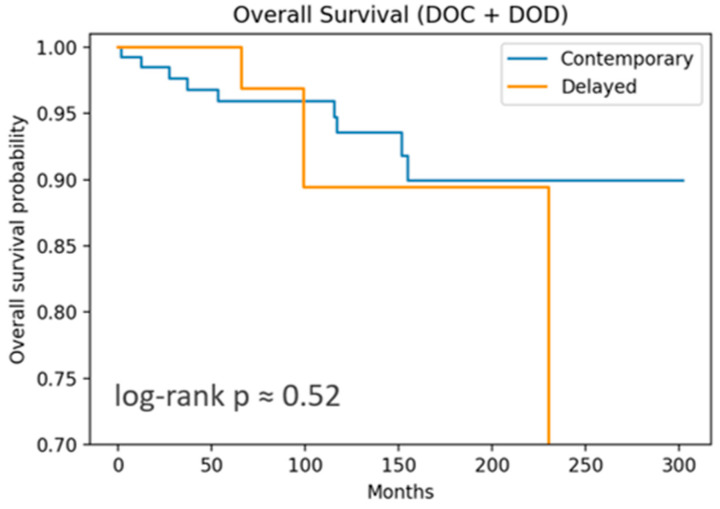
Overall survival analysis comparing contemporary versus delayed treatment approaches. Kaplan–Meier survival curves demonstrate overall survival (OS) probabilities over time (measured in months) for patients receiving contemporary treatment (blue line) versus delayed treatment (yellow line). Events for OS were died of other cause (DOC) and died of disease (DOD). During the follow-up period, 9 events occurred among cLND patients and 3 events in the delayed group. OS did not reach a statistically significant difference (log-rank *p* = 0.52).

**Figure 2:**
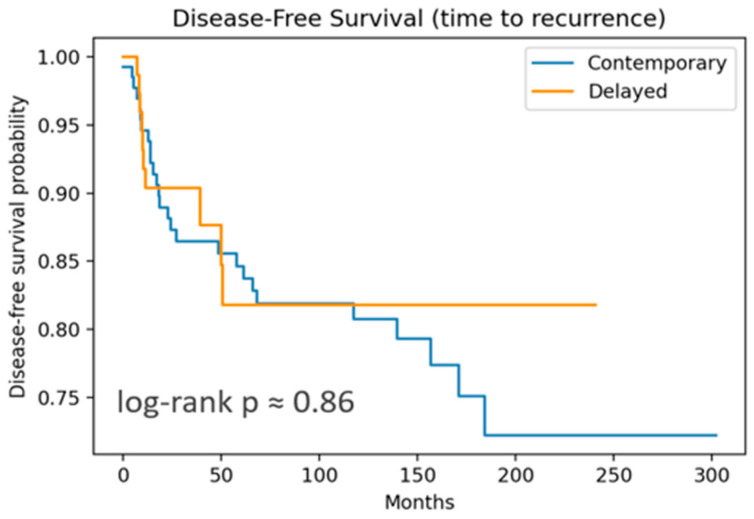
Disease-free survival analysis comparing contemporary versus delayed treatment approaches. Kaplan–Meier curves illustrate disease-free survival (DFS) probabilities over time (measured in months) for patients receiving contemporary treatment (blue line) versus delayed treatment (yellow line). Disease-free survival was defined as the time from treatment initiation to disease recurrence. Disease recurrences were registered in 27 patients for cLND and 10 for dLND. The difference was not statistically significant (log-rank *p* = 0.86).

OS included 12 events, and DFS, 37 recurrences. Both OS and DFS showed no significant differences by log-rank test. DSS curves (Figure 1 and Figure 2) are nearly flat due to the very low number of disease-specific deaths (three events).

For OS, both the cLND and dLND groups demonstrated excellent long-term outcomes. At 1 year, both groups exceeded 99% survival. At 5 years, contemporary OS remained at 96% versus 100% in dLND. At 10 years, contemporary OS was 94%, versus 89% in the delayed group. The absolute difference is only 5% and not statistically significant (log-rank *p* = 0.52). This suggests that the timing of LND—whether performed at the same time as thyroidectomy or as a delayed procedure—did not impact OS in this cohort.

For DFS, both groups showed good outcomes but with more events than OS, as expected. From 3 to 10 years, DFS converges towards 82–87%, showing a small separation early in the follow-up period. Differences in DFS did not achieve statistical significance (log-rank *p* = 0.86), suggesting that the timing of LND does not substantially impact the recurrence risk or survival.

In the univariable analysis for OS, only age significantly raised the hazard of death (DOC + DOD) by about 10% (HR 1.10, *p* < 0.001) per year. No other covariate reached significance. For disease-free survival ECE, the number of pathologic lymph nodes associated with higher recurrence risks (HR 4.0, *p* ≈ 0.001; HR 1.04 per node, *p* < 0.00001) was reconfirmed with the multivariable model.

Finally, in the weighted univariable and multivariable models, age was reconfirmed to be significantly associated with OS (HR ≈ 1.11, *p* < 0.001). Similarly, the total metastatic lymph node count (aHR ≈ 1.04 per node; *p* ≈ 0.005) and ECE (aHR ≈ 3.2; *p* ≈ 0.007) were reconfirmed as major risk factors for recurrences.

### 3.2. Complication Outcomes

**Figure 3:**
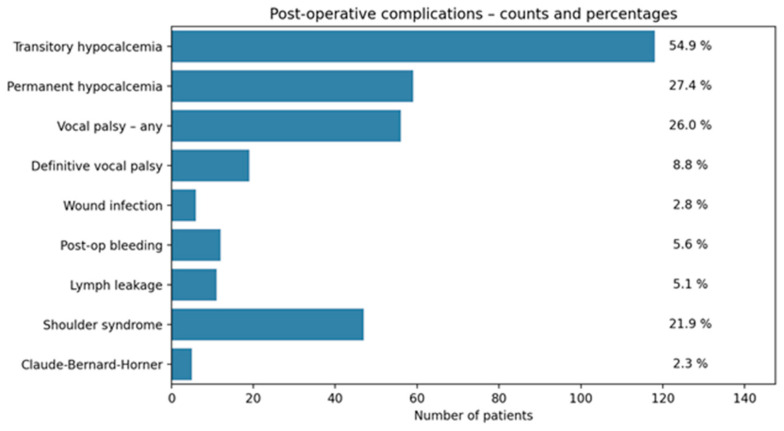
Distribution of postoperative complications in the overall cohort (*n* = 215). Transient hypocalcemia was the most frequent event (54.9%), followed by permanent hypocalcemia (27.4%) and vocal fold palsy (any severity, 26.0%). Definitive vocal palsy occurred in 8.8% of patients. Shoulder syndrome affected 21.9%, while other complications, such as postoperative bleeding, lymphatic leakage, wound infection, and Claude Bernard–Horner syndrome, were less common (<6%).

Among the 215 patients included in the study, transient hypocalcemia occurred in about 55% of patients, while permanent hypocalcemia affected 27%. Vocal fold palsy (both transient and permanent) was observed in 26%, though only 9% progressed to a definitive palsy after 6 months from surgery. Shoulder syndrome was reported in 22%, while Claude Bernard–Horner syndrome, postoperative bleeding, lymphatic leakage, and surgical site infections were less frequent (2–6%) (Figure 3).

Crude comparisons between dLND and cLND revealed several differences. Although transient hypocalcemia was more frequent after dLND (RR ≈ 1.40, *p* ≈ 0.009), permanent hypocalcemia was less frequent in dLND (RR ≈ 0.44. 95% CI 0.24–0.76, *p* = 0.004).

More interestingly, postoperative vocal fold palsy (RR ≈ 0.47. 95% CI 0.19–0.69, *p* = 0.002) and definitive palsy (RR ≈ 0.10. 95% CI 0.01–0.6, *p* = 0.002) were both markedly lower in patients undergoing delayed surgery.

No meaningful differences were detected for bleeding, lymphatic leakage, shoulder syndrome, or Claude Bernard–Horner syndrome; many *p*-values defaulted to 1.00 due to sparse events.

IPTW analysis confirmed these associations, which eventually became more pronounced.

In univariable logistic regression analyses, ECE in VI level lymph nodes emerged as the main significant predictor of postoperative vocal fold palsy, conferring more than a threefold increase in odds (OR ≈ 3.4, 95% CI: 1.7–6.9, *p* = 0.001). pT showed a borderline association, with each incremental stage associated with a roughly 50% increase in risk (OR ≈ 1.5, *p* ≈ 0.07). No individual covariate demonstrated a significant association with the occurrence of permanent hypocalcemia.

In the IPTW-weighted multivariable model focusing on the outcome “vocal palsy”, surgery timing remained a strong independent predictor. Patients undergoing dLND had a significantly lower risk compared to those receiving cLND (OR ≈ 0.25, 95% CI: 0.11–0.55, *p* = 0.001). ECE retained its role as the most powerful risk factor, associated with nearly a fourfold increase in odds (OR ≈ 3.8, 95% CI: 1.8–7.9, *p* < 0.001).

The association between pT stage and vocal palsy persisted as a nonsignificant trend (OR ≈ 1.55, *p* = 0.065), suggesting a possible gradient of risk without definitive statistical support.

OS, DFS, and most complications did not differ significantly between the two surgical approaches between 2013 and 2024. The only statistically significant difference was confirmed to be vocal palsy, which was less frequent in the delayed group (OR 0.26, 95% CI 0.10–0.68, *p* = 0.006), showing the potential independent protective role of the staged procedure.

In our cohort, bilateral neck dissection was performed in a limited subset of patients: 19 in the cLND group and 6 in the dLND group. Regarding postoperative vocal fold palsy, 7 out of 19 patients (36.8%) in the bilateral cLND group experienced complications. Notably, two of these patients developed bilateral vocal fold palsy requiring tracheotomy. In contrast, only one out of six patients (16.7%) in the bilateral dLND group experienced temporary vocal palsy. As for definitive hypocalcemia, it occurred in six patients (31.6%) in the cLND group, while only one patient (16.7%) in the dLND group was affected.

## 4. Discussion

For well-differentiated thyroid cancer, surgery is the gold standard treatment, including lobectomy or total thyroidectomy based on patient risk stratification. Many authors recommend unilateral lobectomy for patients with low-risk papillary carcinoma due to the low mortality and recurrence rates. At the same time, total thyroidectomy should be performed if postoperative iodine-131 treatment is required [1,2].

Although prophylactic CND remains a debated issue and is not universally adopted, some high-volume tertiary care centers, including ours, support its use in selected patients with papillary thyroid cancer. This approach is based on the non-negligible prevalence of occult central compartment lymph node metastases and the generally low risk of complications reported in experienced hands. Moreover, prophylactic CND may improve pathological staging and guide the decision-making process for adjuvant radioactive iodine therapy [9,10].

Conversely, LND is only justified in a therapeutic setting according to most guidelines [2,17,18,19]. Total thyroidectomy and contemporary LND have been shown to share higher rates of adverse events such as hypocalcemia, recurrent laryngeal nerve paralysis, and finally bleeding and neck hematoma, requiring tracheotomy in the worst life-threatening scenarios [11].

In this context, we emphasize the importance of adequate information about the benefits and risks of total thyroidectomy and contemporary LND. Therefore, since 2013, we have been referring selected patients with thyroid cancer and lateral neck metastases to a modified therapeutic pathway, including total thyroidectomy and CND, followed by delayed LND 4–6 weeks after primary surgery. By reducing the extent of the resection and separating the thyroid/central compartment from the lateral compartment, we wanted to test whether total thyroidectomy followed by delayed LND could guarantee similar oncological results. Secondly, the avoidance of direct communication between the central compartment and the lateral neck spaces could reasonably be associated with lower postoperative complication rates.

In our comprehensive analysis of a cohort of 215 patients with differentiated thyroid carcinoma, we investigated the impact of surgical timing on both oncological outcomes and postoperative complications. Specifically, we compared cLND versus dLND.

Our survival analysis demonstrated no significant difference in OS or DFS between the two groups (Log-rank *p* > 0.05), suggesting that the timing of the lateral neck dissection does not compromise oncologic control. These findings challenge the long-standing assumption that a single, immediate, comprehensive surgical approach is superior. Rather, they support the idea that patient and tumor-related factors, more than surgical timing, drive long-term outcomes. In particular, age at surgery emerged as the predominant predictor of OS, while two independent prognostic factors for DFS were identified: the total number of pathological lymph nodes (levels VI + II–V) and ECE.

Notably, surgical timing was not independently associated with DFS, although a nonsignificant trend was observed favoring delayed surgery (HR ≈ 0.42, *p* ≈ 0.08). This further strengthens the hypothesis that the biological aggressiveness of disease rather than the exact timing of surgery is what might influence recurrence patterns.

The identification of ECE and nodal burden as dominant predictors of recurrence has meaningful clinical implications. Patients with ECE were found to have a significantly higher risk of disease recurrence, regardless of the surgical approach. Likewise, the total number of metastatic lymph nodes was strongly associated with recurrence risk. These findings are consistent with the established evidence that nodal status represents a high-risk feature and should be used to guide more intensive treatment strategies, including adjuvant radioactive iodine therapy and closer postoperative surveillance [3,20].

Importantly, the biological significance of major nodal involvement and ECE extends beyond mere anatomical considerations. These features might reflect a more aggressive tumor biology, characterized by enhanced local invasiveness and a greater risk of regional and distant recurrence, as also emphasized by the American Thyroid Association guidelines [18].

We acknowledge that the present study did not extend survival analyses to pT4 tumors. This limitation stems from the composition of our cohort, as most pT4 patients were managed with cLND in earlier years, and few were treated with a delayed approach in recent clinical practice. This is in line with recent trends in the literature, where increased detection of pT1–pT3 tumors has been attributed to widespread screening and early diagnosis in high-income healthcare systems [21].

Beyond oncologic endpoints, our study revealed important differences in complication patterns between the two surgical strategies. Overall complication rates were comparable between cLND and dLND with previously published series [7,22,23,24,25,26,27,28]. Nevertheless, we observed a notably higher rate of permanent vocal fold paralysis in the contemporaneous group. This difference likely reflects the technical complexity of performing an extensive one-stage procedure, which may increase the risk of recurrent nerve trauma due to cumulative manipulation and prolonged surgical time.

The elevated incidence of definitive vocal cord palsy in our cohort is not unexpected given our high-volume oncologic setting. For instance, a study focusing on medullary thyroid cancer reported a 16.5% rate of vocal cord paralysis, significantly higher than in general thyroid surgery populations [27]. Similarly, a large-scale series of 5670 total thyroidectomies for well-differentiated thyroid carcinoma reported a 9.5% complication rate due to vocal fold paralysis [28]. These figures support our observation that patients with advanced nodal disease undergoing oncologic thyroid surgery are at higher risk of nerve-related complications, particularly when lateral neck dissection is performed in a one stage procedure.

We also examined the potential role of tumor size, indirectly measured through pT classification, as a risk factor for vocal fold palsy. In our multivariable analysis, pT stage showed a borderline but not statistically significant association with vocal palsy risk (OR ≈ 1.55, *p* = 0.065). However, we recognize that pT is an imperfect surrogate for tumor volume or anatomical complexity, and this likely contributed to the imprecision of the observed effect. While the trend suggests a possible link between increasing tumor burden and surgical difficulty, particularly in dissecting the recurrent nerve, further research using more precise volumetric measures is warranted.

By contrast, ECE in VI level lymph nodes emerged as a strong and independent predictor of vocal fold palsy in our analysis. The presence of ECE was associated with an OR of 3.8 (95% CI: 1.8–7.9, *p* < 0.001), highlighting the increased complexity of lymph node dissection in these cases. This reinforces the idea that vocal fold paralysis may result not only from technical execution but also from biologically aggressive disease that involves or distorts the anatomical plane around the recurrent nerve.

Intraoperative nerve monitoring (IONM) provides valuable insights when integrated into multidisciplinary board discussions and surgical planning, particularly in complex scenarios such as bilateral neck dissection. Based on our findings—highlighting an independent association between surgical timing and vocal fold palsy—we believe IONM plays a more strategic role in guiding surgical staging decisions than in directly reducing nerve injury. Specifically, early signal loss (LOS) during thyroidectomy, particularly on the side of the primary tumor, may prompt a delayed approach to contralateral surgery to prevent bilateral recurrent laryngeal nerve paralysis. In our experience, the risk of nerve damage is more often driven by the biological aggressiveness of the tumor and the surgical complexity of these high-risk patients, rather than solely by the absence of IONM.

Although the small sample size of patients who underwent bilateral neck dissection prevents definitive conclusions, the crude complication rates suggest a potential advantage of the delayed approach even in patients undergoing bilateral neck dissection. These findings are consistent with the overall trend observed in our cohort and further support a more cautious and individualized approach in bilateral pN1b cases.

Most compellingly, our data supported the protective role of dLND. The delayed approach was associated with a significantly lower risk of vocal fold palsy, even after adjustment for confounders.

Different authors have addressed how extensive one-stage procedures may increase the risk of complications, particularly recurrent nerve injury, due to operative fatigue and prolonged procedural time. Furthermore, thyroidectomy durations exceeding 120 min independently doubled the risk of hypoparathyroidism, supporting concerns about surgeon fatigue and ischemic injury in lengthy surgeries [5,29]

We hypothesize that separating the thyroidectomy and neck dissection phases may reduce the cumulative operative stress on the recurrent laryngeal nerve. Prolonged, one-stage procedures may increase the risk of neuropraxia due to sustained retraction or thermal injury, especially in cases of bulky nodal disease.

Conversely, the two-step strategy may offer physiological and practical advantages: it enables the surgeon to maintain a sharper technical focus during each phase of the operation, particularly during the thyroidectomy when nerve identification and preservation are most critical. By splitting a major oncologic procedure into two distinct, more focused operations, even highly skilled surgeons may optimize performance and reduce the likelihood of inadvertent nerve trauma.

Ultimately, we are fully aware that a two-stage surgical strategy implies two separate hospital admissions and repeated exposure to general anesthesia. These aspects represent important considerations, particularly in terms of healthcare logistics and patient experience.

Nevertheless, in light of the significantly lower risk of permanent vocal fold paralysis observed with delayed lateral neck dissection, we believe that this approach deserves consideration, especially for medically complex or frail patients. Rather than suggesting a definitive change in practice, we recommend dLND as an evidence-supported alternative to be discussed in a shared decision-making process, tailored to individual patient characteristics and preferences.

Our findings raise additional questions regarding postoperative quality of life, particularly in patients affected by definitive vocal fold palsy and hypocalcemia. Beyond functional impairment, definitive vocal fold paralysis may entail prolonged rehabilitation, repeated evaluations by speech language pathologists, and economic consequences including work limitations and indirect healthcare costs [30]. Similarly, the burden of permanent hypoparathyroidism should not be underestimated, especially considering long-term calcium and vitamin D supplementation requirements [31].

Future studies should incorporate patient-reported outcomes and health economic evaluations to fully assess the long-term burden of complications such as dysphonia. As a natural continuation of this work, we plan to implement a cost-effectiveness analysis comparing dLND and cLND, integrating functional outcomes and rehabilitation costs into the overall assessment of these two surgical strategies.

### Limitations

First, the retrospective, single-center design inherently introduces potential selection bias and limits generalizability to other institutions.

Although IPTW methodology and E-value analysis strengthened our comparative analyses, the potential for residual confounding and the lack of external validation in multicenter cohorts suggest that our findings should be interpreted as hypothesis-generating rather than definitive evidence. As a retrospective study, this work indirectly assessed the role of tumor size in driving complications through pT stage; nevertheless, it was not possible to evaluate homogeneously tumor location in the thyroid gland, nor the volume of the nodule directly.

All patients were in the pN1b stage and received postoperative RAI therapy. While we assessed nodal burden quantitatively and qualitatively, detailed RAI parameters were unavailable as many patients completed treatment externally. However, universal RAI administration eliminates RAI as a confounder, enhancing the validity of our surgical timing comparisons.

Despite these limitations, our study provides valuable hypothesis-generating evidence regarding surgical timing in lateral neck dissection. The robust statistical methodology, including IPTW analysis and sensitivity tests, strengthens confidence in the observed associations while acknowledging the need for external validation.

## 5. Conclusions

Delayed lateral neck dissection appears to be a safe and effective alternative to traditional single-stage surgery in patients with pT1–pT3 differentiated thyroid carcinoma.

While oncologic outcomes remain comparable, the significantly lower risk of definitive vocal fold paralysis associated with the delayed approach marks a meaningful step toward balancing oncologic radicality with functional preservation.

These findings advocate for the integration of delayed surgical strategies into standard treatment algorithms, allowing surgeons to tailor their approach based on individual patient and tumor characteristics. Delayed neck dissection offers a pragmatic, evidence-based option for selected patients, potentially improving quality of life without compromising disease control.

Future prospective studies will be critical to validate these results, refine patient selection criteria, and optimize surgical planning in this complex and evolving clinical landscape.

## Figures and Tables

**Table 1:** Baseline clinical–demographic characteristics of patients undergoing contemporary (one-stage) versus delayed (two-stage) lateral neck dissection for differentiated thyroid cancer.

Clinical and Demographic Characteristics	Contemporary	Delayed	*p*-Value
Number (%)	136 (100%)	79 (100%)	
Age (mean ± SD)	42.8 ± 15.1	45.6 ± 15.2	0.131
Female	77 (56.6%)	49 (62.0%)	0.475
Male	59 (43.4%)	30 (38.0%)	0.475
CCI 0	106 (77.9%)	58 (73.4%)	0.507
CCI ≥ 1	30 (22.1%)	21 (26.6%)	0.507
pT1	96 (70.6%)	59 (74.7%)	
pT2	23 (16.9%)	14 (17.7%)	
pT3	17 (12.5%)	6 (7.6%)	0.533
Path LN VI (mean ± SD)	4.4 ± 4.7	5.8 ± 6.9	0.084
Path LN II–V (mean ± SD)	5.8 ± 4.3	8.7 ± 9.7	0.172
NED	118 (86.8%)	69 (87.3%)	
AWD	9 (6.6%)	7 (8.9%)	
DOC	7 (5.1%)	2 (2.5%)	
DOD	2 (1.5%)	1 (1.3%)	0.760

CCI = Charlson Comorbidity Index. pT = pathological primary tumor stage (AJCC 8th edition). LN = lymph node. NED = no evidence of disease at last follow-up. AWD = alive with disease. DOC = died of other causes. DOD = died of disease.

## Data Availability

The data presented in this study are available on request from the corresponding authors Marta Tagliabue and Rita De Berardinis (marta.tagliabue@ieo.it; rita.deberardinis@ieo.it).

## Supplementary Materials

The following supporting information can be downloaded at: https://www.mdpi.com/article/10.3390/cancers17162649/s1, Supplementary Materials: Robustness and Sensitivity Analyses Supporting the Main Findings.
