# Supplementary material for: Surgical Timing in Thyroid Cancer with Lateral Neck Metastases: Delayed Versus Contemporary Lateral Neck Dissection
Source: Cancers (Basel). 2025 Aug 14;17(16):2649. doi: 10.3390/cancers17162649 (PMC12384942; doi:10.3390/cancers17162649)
Supplement: Supplementary file 1 [file cancers-17-02649-s001.zip › cancers-3776774-supplementary.pdf]

Article

Surgical Timing in Thyroid Cancer with Lateral Neck Metastases: Delayed Versus Contemporary Lateral Neck Dissection

Francesco Chu [1,†], Rita De Berardinis [1,*,†], Marta Tagliabue [1,*], Roberto Bruschini [1], Stefano Filippo Zorzi [1], Marco Federico Manzoni [2], Maria Cecilia Mariani [2], Enrica Grosso [1], Gioacchino Giugliano [1,‡] and Mohssen Ansarin [1,‡]

Supplementary Materials

Robustness and Sensitivity Analyses Supporting the Main Findings

1. Inverse Probability of Treatment Weighting (IPTW) and Covariate Balance

To address potential confounding, inverse probability of treatment weighting (IPTW) was used. The propensity model included: age, sex, pathological T stage (pT), multifocality, extracapsular extension (ECE), and number of pathological lymph nodes in level VI and levels II–V. After weighting, standardized mean differences for all covariates fell below the ±0.10 threshold, indicating excellent balance.

2. Survival Model Robustness

Both Cox proportional hazards and Weibull accelerated failure time (AFT) models were fitted for overall survival (OS) and disease-free survival (DFS). Schoenfeld residuals confirmed no significant violations of the proportional hazards assumption. Weibull AFT models produced consistent results with the Cox model, validating robustness.

3. Weight Trimming and Propensity Score Matching

IPTW weights were trimmed at the 1st–99th and 5th–95th percentiles. Results remained stable. Two additional matching approaches—2:1 matching and full-sample kernel matching—confirmed the DFS effect with hazard ratios between 0.40 and 0.44.

4. Doubly Robust Estimation and Bootstrap Resampling

Doubly robust regression models confirmed the protective effect of delayed surgery. Bootstrap resampling with 1,000 replicates yielded bias-corrected confidence intervals consistent with the main estimates.

5. Influence Diagnostics

DFBETAs showed no single patient had undue influence on the effect estimates. Maximum DFBETA values for key predictors were < 0.23, within acceptable thresholds.

6. E-values for Unmeasured Confounding

E-values were calculated to quantify the strength of unmeasured confounding needed to negate observed associations. For the effect of delayed surgery on vocal fold palsy (OR = 0.25), the point E-value was 7.46 and the lower-bound E-value was 3.33. For ECE (OR = 3.8), the E-value was 7.04 (lower-bound = 3.02), supporting robustness against unmeasured bias.

7. Restricted Mean Survival Time (RMST)

RMST analysis at 5 years showed a 2.7-month advantage in recurrence-free survival for the delayed group (95% CI −0.5 to 6.0 months, $p \approx 0.10$). Although not statistically significant, this complements the Cox and AFT results.
